# Inadequacies in the habitual nutrient intakes of patients with metabolic syndrome: a cross-sectional study

**DOI:** 10.1186/s13098-016-0147-3

**Published:** 2016-04-14

**Authors:** Aline Tuane Oliveira da Cunha, Hermilla Torres Pereira, Sephora Louyse Silva de Aquino, Cristiane Hermes Sales, Karine Cavalcanti Maurício Sena-Evangelista, Josivan Gomes Lima, Severina Carla Vieira Cunha Lima, Lucia Fatima Campos Pedrosa

**Affiliations:** Post-Graduate Program in Health Sciences, Federal University of Rio Grande do Norte, Av. General Cordeiro de Farias, s/nº- Petrópolis, Natal, RN CEP: 59012-570 Brazil; Post-Graduate Program in Nutrition, Federal University of Rio Grande do Norte, Av. Sen. Salgado Filho 3000, Lagoa Nova, Campus Central, Natal, RN CEP: 59078-970 Brazil; Department of Nutrition, School of Public Health of University of São Paulo, Av. Doutor Arnaldo, 715, Sumaré, São Paulo, SP CEP: 01246904 Brazil; Department of Nutrition, Federal University of Rio Grande do Norte, Av. Sen. Salgado Filho 3000, Lagoa Nova, Campus Central, Natal, RN CEP: 59078-970 Brazil; Departamento de Medicina Clínica, Universidade Federal do Rio Grande do Norte, Av. Nilo Peçanha, 620, Petrópolis, Natal, RN CEP: 59010-180 Brazil

**Keywords:** Metabolic syndrome, Food intake, Nutrients, Nutritional requirements, EAR

## Abstract

**Background:**

Dietary factors are important environmental factors associated with the prevalence of metabolic syndrome (MS). The objective of this study was to assess the habitual nutrient intakes of patients with MS.

**Methods:**

A cross-sectional study included 103 patients (82 % women) with MS seen at the endocrinology outpatient clinic of Hospital Universitario Onofre Lopes. Habitual nutrient intake data were collected at two 24-h dietary recalls. Macronutrient intake adequacies were classified according to the I Brazilian guidelines for the diagnosis and treatment of metabolic syndrome. The prevalence of inadequate micronutrient intake was estimated using the estimated average requirements (EAR) cut-point method after adjusting for intra- and interpersonal variances and energy.

**Results:**

The mean energy intake of the included patients was 1523.0 ± 592.2 kcal/d, higher in men (1884.0 vs. 1441.5 kcal/d in women; *p* = 0.003). The recommended percentage protein intake was exceeded in both women and men (18 % in women and 19 % in men). Although men consumed more fiber (18.8 vs. 13.3 g/d in women; *p* = 0.011), their intake was still inadequate. Women consumed more fat (47.6 vs. 41.3 g/d in men; *p* = 0.007). The prevalence of inadequate vitamin D and calcium intakes exceeded 80 % in both men and women and across all age groups. The same was observed for magnesium in men and women aged more than 30 years. The prevalence of inadequate vitamin E, riboflavin, and zinc intakes in men ranged from 50 to 75 %. The prevalence of inadequate vitamin A, vitamin C, thiamin, vitamin B6, copper, and selenium intakes in men and women was less than 50 %.

**Conclusions:**

Patients with MS had high protein intake, low fiber intake, and high a prevalence of inadequate vitamin D, magnesium, and calcium intakes.

## Background

Metabolic syndrome (MS) is a complex disorder represented by a set of cardiovascular risk factors that include hyperglycemia, hypertriglyceridemia, low levels of high-density lipoprotein cholesterol (HDL-c), elevated waist circumference, and high blood pressure [1].

The global prevalence of MS in adults ranges from 20 to 25 % [2], and in Brazil, from 4.9 to 65.3 %, including urban and rural areas and indigenous populations [3]. The prevalence of MS in adults aged 20–59 years was 4.5 % and increased with age, from 1.3 % in adults aged 20–29 years to 5.6 % in those aged 30–39 years and 26.3 % in those aged ≥40 years [4].

MS has a multifactorial etiology that depends on interactions between metabolic, genetic, and environmental factors. Among the environmental factors, diet is one of the main risk factors associated with the increased prevalence of MS [5]. Dietary intervention is an important strategy for the prevention and control of MS [6].

In recent years, the effects of isolated foods and dietary patterns have been investigated in the development of MS [7–10]. Dietary patterns consisting of high consumption of red and/or processed meat, refined grains, and fried foods are directly associated with an increased risk of MS [8, 9], while the high consumption of vegetables, fruits, and fish has provided a protective effect [9–11].

As with dietary patterns, studies on isolated nutrients have also demonstrated an influence in the development of MS, thus highlighting the importance of an adequate diet in terms of both macro- and micronutrients. Saturated fat intake greater than 10 % of the total caloric value indicated a risk for MS in Brazilian adults [11]. Low intake of antioxidant nutrients such as vitamin C, selenium, and zinc might also predispose the development of MS [12, 13]. Negative significant correlation coefficients were observed among dietary fat, energy, protein intake, and serum antioxidant state. It is well known that oxidative stress plays a role in the pathogenesis of MS components [12].

Deficiency of 25(OH)D has been linked to both the etiology of MS and the isolated components [14]. A meta-regression model showed that dietary magnesium intake is significantly and inversely associated with the risk of metabolic syndrome. These findings are consistent with clinical studies that demonstrated that magnesium oral supplementation improved metabolic profile and blood pressure in metabolically obese, normal-weight individuals [15, 16].

National dietary surveys performed in the Brazilian adult population have found unbalanced diet in proportion to intake of energy and macronutrients such as low-carbohydrate and high-fat diet. In addition, the intake of calcium, vitamin D, and vitamin E was inadequate in more than 80 % of both men and women [17, 18]. However, studies using statistical methods for known deficiency of nutrient intakes in patients with MS are still scarce in the literature.

Hence, the present study aimed to assess the habitual nutrient intakes of patients with MS. The identification of possible dietary inadequacies will be useful for health care professionals to prevent and treat this disease.

## Methods

### Study design

This analytical, cross-sectional study was conducted at the Hospital Universitario Onofre Lopes (HUOL), located in Natal, RN, Northeast Brazil. This study was approved by the hospital’s Research Ethics Committee under protocol number CAAE no. 13699913.7.0000.5292. All participants signed an informed consent form.

### Population and the study cohort

The population of the study consisted of adults and elderly adults of both sexes seen at the endocrinology outpatient clinic of the HUOL.

The inclusion criteria were age 19–80 years and the presence of MS according to the criteria of the National Cholesterol Education Program—Third Adult Treatment Panel (NCEP-ATP III) [1]. The cut-off point used by the study for fasting glucose was 100 mg/dL. The diagnosis of MS was based on the presence of at least three of the following: waist circumference >102 cm in men and >88 cm in women; triglyceride level ≥150 mg/dL; HDL-c level <40 mg/dL in men and <50 mg/dL in women; systolic blood pressure ≥130 mmHg and/or diastolic blood pressure ≥85 mmHg or use of antihypertensive drugs; and fasting glucose ≥100 mg/dL or use of oral antidiabetic drugs.

The exclusion criteria were type 1 diabetes mellitus; type 2 diabetes mellitus with insulin use; use of glucocorticoids in the past 3 months; use of calcium or vitamin D supplements in the past 30 days; and use of anticonvulsants or rifampicin. Pregnant and nursing patients and patients with kidney failure were also excluded.

Between June 2013 and May 2014, 1500 medical records were screened. A total of 140 individuals met the inclusion criteria, 714 had one or more exclusion criteria, and 646 did not have a diagnosis of MS. Of the 140 eligible patients, 103 were finally included in the study, representing a sample loss of 26 %.

First, we collected the anthropometric measurements of the patients and administered the 24-h recall (24-HR) and a questionnaire on lifestyle and health status. We then conducted biochemical tests and scheduled a second administration of the 24-HR after 30–45 days.

### Anthropometric assessment

The body mass index (BMI) of adults (<60 years) was classified according to the recommendations of the World Health Organization (WHO) [19]. The BMI of elderly participants (i.e. ≥60 years) was classified according to the criteria given by Lipschitz [20].

Waist circumference was measured at the midpoint between the last rib and the iliac crest by using an inelastic tape measure. Waist circumference was considered elevated when it was >88 cm in women and >102 cm in men [1].

### Blood pressure

Blood pressure was measured as recommended by the VI Brazilian guidelines on hypertension [21]. High blood pressure was defined as systolic blood pressure ≥130 mmHg and/or diastolic blood pressure ≥85 mmHg.

### Biochemical tests

Blood samples were collected via venipuncture after an overnight fast of 10–12 h. Blood glucose, triglycerides, and HDL-c levels were determined via colorimetry by using automatic equipment and kits from Wiener^®^ lab 2000 (Rosario, Argentina).

### Lifestyle assessment

Alcohol intake was classified according to the type of alcoholic beverage and the number of servings consumed in a month. A serving was defined as a can of beer; a glass of wine; or a shot of rum, vodka, whisky, or any other distilled beverage [22].

The smoking status was classified as follows: nonsmoker, when the individual had smoked fewer than 100 cigarettes over his lifetime; ex-smoker, when the individual had smoked at least 100 cigarettes over his lifetime but had not smoked in the past year; and smoker, when the individual had smoked more than 100 cigarettes over his lifetime and continued to smoke [23].

Physical activity was measured by using the International Physical Activity Questionnaire-short form (IPAQ-SF), validated for Brazil by Matsudo et al. [24]. Individuals were classified as inactive, irregularly active (categories A and B), active, and very active.

### Food intake assessment

Habitual food intake was investigated by using two 24-HR at the following times: (1) every alternate day, except weekends and holidays to avoid collecting atypical data; and (2) distinctive times throughout the month, considering the family’s purchasing power.

Photographs of utensils and containers were used to identify the food-serving items and quantify serving sizes, classified as small, average, or large. The foods were converted into nutrients by the software Virtual Nutri Plus^®^ 2.0 (São Paulo, Brazil). New preparations and foods were added to the database as necessary, along with their nutritional composition, from The Brazilian food composition table [25] and the United States Department of Agriculture (USDA) [26] database, whichever was more appropriate. Nutritional information collected from processed food labels was also included. Participants with reported energy intakes <500 or >5000 kcal were excluded [27]. Energy, macronutrient, and fiber intake adequacies were classified as recommended by the I Brazilian guidelines for diagnosing and treating metabolic syndrome [5].

The prevalence of inadequate micronutrients intake was estimated according to sex and age by using the estimated average requirements (EAR) cut-point method [28–31], except for iron, whose prevalence was calculated via the probability approach [32]. The prevalence of inadequate intake of each micronutrient was estimated considering the proportion of individuals with intake below the EAR value. Potassium intake was evaluated based on adequate intake (AI). The percentage of individuals whose sodium intake exceeded the tolerable upper intake level (UL) was also calculated [33].

### Statistical analysis

Student’s *t* test was used to compare the dietary variables, expressed as means and standard deviations. The significance level was set at 5 %. The Kolmogorov–Smirnov test was used to verify data distribution symmetry. Nutrients with asymmetric distribution were converted into natural logarithms and retested for symmetry. After the logarithmic transformation, only vitamin B12 intake remained asymmetrically distributed, so it was excluded from the analysis. Continuous variables with symmetric distribution were expressed as means and standard deviations; variables with asymmetric distribution, as median and interquartile ranges (Q_25_–Q_75_); and categorical variables, as absolute and relative frequencies.

Because of dietary data variability, intrapersonal variability was adjusted by the method developed by Nusser et al. [34]. Later, the results were adjusted for energy [35].

One-way analysis of variance (ANOVA) was used to determine intrapersonal variability, and estimates of intra- and interpersonal variances were based on the resulting quadratic means [34]. In order to control the confounding factors inherent to total energy intake and remove external variables, the data were adjusted for energy by using the residue method [35]. Energy was included as an independent variable, and absolute nutrient intake, as a dependent variable.

## Results

The most frequent MS components were high blood pressure (78 %), waist circumference (75 %), and altered fasting blood glucose (73 %). Characteristics of patients with MS are shown in Table 1. Mean patient age was 50 ± 13.2 years, and 82 % (n = 84) were women. Participants’ lifestyles revealed that most did not consume alcoholic beverages, did not smoke and were active.

**Table 1:** General characteristics of patients with metabolic syndrome by sex

Variables	Total (*n* = 103)	Women (*n* = 84)	Men (*n* = 19)
Age (years)^a^	50 (13.2)	49 (13.4)	57 (10.4)
Body mass index (kg/m^2^)^a^	33.5 (7.3)	34.2 (7.5)	30.5 (5.6)
Waist circumference (cm)^a^	106.7 (13.6)	106.9 (13.6)	105.9 (14.0)
HDL-cholesterol (mg/dL)^a^	40.4 (12.9)	41.0 (13.8)	37.6 (7.7)
Systolic blood pressure (mm/Hg)^a^	131.1 (10.4)	131.4 (10.6)	129.8 (9.2)
Diastolic blood pressure (mm/Hg)^b^	85.0 (80.0–90.0)	85.0 (80.0–90.0)	85.0 (80.0–90.0)
Triglycerides (mg/dL)^b^	162.0 (119.0–244.0)	162.5 (130.0–272.5)	162.0 (100.0–191.0)
Fasting blood glucose (mg/dL)^b^	109.0 (102.0–136.0)	109.0 (102.0–131.8)	109.0 (104.0–163.0)
Smoking status (%)			
Non-smoker	50	51	42
Ex-smoker	40	38	47
Smoker	10	11	11
Alcohol intake (%)			
No intake	81	86	63
1–4 servings/month	6	5	11
>4 servings/month	13	9	26
Level of physical activity (%)			
Inactive	5	5	5
Irregularly active	37	36	42
Active	58	59	53

^a^Results expressed as mean (standard deviation)

^b^Median (Q_25_ and Q_75_) or %

The mean energy intake of the cohort was 1523.1 ± 592.2 kcal/d, differing significantly between men (1884.0 kcal/d) and women (1441.5 kcal/d) (*p* = 0.003). Both men and women had a proper percentage of carbohydrate and fat intake, but a high percentage of protein intake (Table 2).

**Table 2:** Energy and macronutrient intakes of patients with metabolic syndrome by sex

Energy/macronutrients/fiber	Total (103)	Women (84)	Men (19)	*p*
Energy (kcal/d)	1523.1 (592.2)	1441.5 (539.2)	1884.0 (691.8)	0.003
Crude carbohydrate				
kcal/d	833.3 (317.9)	786.1 (295.2)	1041.8 (338.5)	0.001
g/d	208.3 (79.5)	196.5 (73.8)	260.5 (84.6)	
% of total calories	*55*	*55*	*56*	
Adjusted carbohydrate				
kcal/d	836.2 (126.4)	832.4 (133.6)	853. 1 (88.7)	0.412
g/d	209.1 (31.6)	208.1 (33.4)	213.3 (22.2)	
% of total calories	*55*	*54*	*56*	
Crude protein				
kcal/d	274.1 (127.4)	255.4 (111.7)	356.7 (159.9)	0.016
g/d	68.5 (31.9)	63.9 (27.9)	89.2 (40.0)	
% of total calories	*18*	*18*	*19*	
Adjusted protein				
kcal/d	275.2 (54.1)	272.3 (50.6)	288.3 (67.4)	0.339
g/d	68.8 (13.5)	68.1 (12.7)	72.1 (16.9)	
% of total calories	*18*	*18*	*19*	
Crude total fat				
kcal/d	415.7 (227.9)	399.9 (218.7)	485.5 (260.1)	0.140
g/d	46.2 (25.3)	44.4 (24.3)	54.0 (28.9)	
% of total calories	*27*	*27*	*25*	
Adjusted total fat				
kcal/d	417.8 (83.2)	428.2 (78.7)	371.9 (89.0)	0.007
g/d	46.4 (9.2)	47.6 (8.7)	41.3 (9.9)	
% of total calories	*27*	*28*	*25*	
Crude fiber				
g/d	14.3 (8.5)	13.3 (7.5)	18.8 (11.2)	0.011
Adjusted fiber				
g/d	12.7 (3.8)	12.6 (3.7)	13.2 (4.7)	0.614

Results expressed as mean (standard deviation). The crude values refer to the absolute intakes of the study patients, and the adjusted values refer to the results after adjusting for intrapersonal variability [25] and energy [26]

Reference values according to the I Brazilian Guideline for Diagnosing and Treating Metabolic Syndrome, 2005

Carbohydrate: 50–60 % of total energy intake

Protein: 15 % of total energy intake

Total fat: 25–35 % of total energy intake

Fiber: 20–30 g/d

Men consumed more carbohydrates and proteins (crude values) (260.5 vs. 196.5 g/d in women; *p* = 0.001 and 89.2 vs. 63.9 g/d in women; *p* = 0.016, respectively), but women consumed more total fat (adjusted value) (47.6 vs. 41.3 g/d in men; *p* = 0.007). Although men consumed more fiber (crude value) (18.8 vs. 13.3 g/d in women; *p* = 0.011), both men and women had inadequate fiber intake (Table 2).

The prevalence of inadequate vitamin D, magnesium, and calcium intakes was high, ranging from 68 to 100 % in both men and women (Tables 3 and 4). Additionally, men had a high prevalence of inadequate riboflavin (52.2 %), vitamin E (60.3 %), and zinc (75.5 %) intakes (Table 4). Approximately 35 % of women had inadequate zinc intake.

**Table 3:** Nutritional recommendations, intake, and prevalence of inadequate micronutrient intakes in women with metabolic syndrome (*n* = 84)

Micronutrients	EAR/AI	Mean	SD	Intake percentiles					% of inadequacy
				10th	25th	50th	75th	90th	
Vitamin A (μg/d)^a^	500	659.8	518.9	263.4	354.8	498.4	760.2	1427.9	37.8
Vitamin C (mg/d)	60	88.5	83.3	27.0	38.9	73.2	109.3	163.1	36.7
Vitamin D (μg/d)	10	2.7	1.3	1.3	1.7	2.4	3.1	4.6	100.0
Vitamin E (mg/d)^b^	12	12.1	6.1	5.6	7.5	10.9	15.7	21.0	49.2
Thiamin (mg/d)	0.9	1.1	0.6	0.6	0.8	1.0	1.2	1.4	37.8
Riboflavin (mg/d)	0.9	1.0	0.4	0.6	0.7	0.9	1.1	1.3	45.2
Vitamin B6 (mg/d)^c^ 19–50 years	1.1	1.4	0.3	1.1	1.3	1.4	1.6	1.8	11.1
>50 years	1.3	1.5	0.2	1.3	1.4	1.5	1.6	1.7	15.4
Niacin (mg/d)^d^	11	17.7	6.7	11.8	13.9	16.7	21.0	24.9	15.9
Phosphorus (mg/d)	580	776.7	232.5	528.2	632.6	757.8	939.5	1060.2	19.8
Magnesium (mg/d)^e^ 19–30 years	255	200.9	114.8	120.4	138.5	163.6	244.4	431.4	68.1
>30 years	265	184.1	51.7	123.2	154.6	177.6	206.9	237.2	94.1
Zinc (mg/d)	6.8	7.8	2.4	5.3	6.2	7.2	8.8	10.8	34.8
Copper (mg/d)	0.7	0.9	0.3	0.7	0.7	0.8	0.9	1.1	25.5
Iron (mg/d)^c,f^ 19–50 years	8.1	11.4	2.3	9.0	10.3	10.8	12.7	13.7	2.0
>50 years	5.0	11.2	1.1	10.0	10.2	11.0	11.9	12.9	7.8
Selenium (μg/d)	45	54.2	28.4	22.9	35.8	50.4	66.8	85.5	37.1
Calcium (mg/d)^c^ 19–50 years	800	454.3	141.8	289.5	342.3	430.2	528.1	659.2	99.3
>50 years	1000	463.3	111.7	330.5	373.3	456.0	542.9	607.3	100.0
Potassium (mg/d)^g^	4700	1706.1	514.0	1110.1	1374.5	1647.0	1945.0	2464.3	–

*EAR* estimated average requirement; *AI* adequate intake

^a^Calculated as retinol activity equivalents

^b^Calculated as α-tocopherol equivalents

^c^19–50 years (*n* = 44); >50 years (*n* = 40)

^d^Calculated as niacin equivalents

^e^19–30 years (*n* = 6); >30 years (*n* = 78)

^f^Inadequate iron intake was calculated by the probability approach

^g^AI value

**Table 4:** Nutritional recommendations, intake, and prevalence of inadequate micronutrient intakes in men with metabolic syndrome (*n* = 19)

Micronutrients	EAR/AI	Mean	SD	Intake percentiles					% of inadequacy
				10th	25th	50th	75th	90th	
Vitamin A (μg/d)^a^	625	652.2	547.7	266.8	352.6	467.7	756.9	1287.3	48.0
Vitamin C (mg/d)	75	99.8	78.7	29.6	58.7	86.6	118.4	151.5	37.8
Vitamin D (μg/d)	10	2.6	1.4	1.2	1.6	2.3	3.1	4.7	100.0
Vitamin E (mg/d)^b^	12	10.6	5.1	4.4	7.2	9.9	14.1	16.9	60.3
Thiamin (mg/d)	1	1.3	0.8	0.7	0.9	1.0	1.3	1.6	33.7
Riboflavin (mg/d)	1.1	1.1	0.5	0.8	0.8	0.9	1.1	1.4	52.8
Vitamin B6 (mg/d)^c^ 19–50 years	1.1	1.5	0.3	1.1	1.2	1.4	1.7	1.8	9.7
>50 years	1.4	1.6	0.5	1.1	1.3	1.5	1.8	2.6	32.3
Niacin (mg/d)^d^	12	21.2	10.9	10.7	14.5	18.4	24.3	36.8	19.8
Phosphorus (mg/d)	580	852.5	229.3	577.5	687.1	829.2	963.1	1128.9	11.7
Magnesium (mg/d)^e^ 19–30 years	330	168.2	36.1	114.1	144.4	162.3	203.9	216.1	100.0
>30 years	350	220.7	68.8	135.4	170.1	202.3	248.5	350.0	97.0
Zinc (mg/d)	9.4	7.3	3.1	4.4	5.3	6.7	8.1	10.4	75.5
Copper (mg/d)	0.7	0.9	0.3	0.7	0.7	0.8	1.0	1.2	23.0
Iron (mg/d)^f^	6	11.7	2.5	9.1	10.6	11.3	12.8	16.1	0.0
Selenium (μg/d)	45	59.8	22.4	30.5	40.8	61.5	75.1	95.6	25.5
Calcium (mg/d)^g^ 19–50 years	800	466.0	123.6	314.6	346.2	450.5	558.7	669.4	99.7
>50 years	1000	639.5	–	–	–	–	–	–	–
Potassium (mg/d)^h^	4700	1817.9	503.8	1350.0	1473.2	1721.3	2058.9	2506.7	–

*EAR* estimated average requirement; *AI* adequate intake

^a^Calculated as retinol activity equivalents

^b^Calculated as α-tocopherol equivalents

^c^19–50 y (*n* = 6); >50 y (*n* = 13)

^d^Calculated as niacin equivalents

^e^19–30 y (*n* = 6); >30 y (*n* = 13)

^f^Inadequate iron intake was calculated by the probability approach

^g^19–50 y (*n* = 18); >50 y (*n* = 1)

^h^AI value

The prevalence of inadequate vitamin A, vitamin C, thiamin, vitamin B_6_, copper, and selenium intakes in men and women was below 50 %. None of the men had an inadequate iron intake. The potassium intake of all patients was below the AI (Tables 3 and 4). The mean sodium intake was high (1964.6 mg/d ± 520.3 in women and 1898.5 mg ± 587.6 in men), demonstrating that more than 70 % of the cohort exceeded the UL.

## Discussion

An initial therapeutic approach to the prevention and treatment of MS involves lifestyle changes. The satisfactory results of the participants’ lifestyles may stem from the fact that these patients were seen and/or followed at a multidisciplinary outpatient clinic, which may have encouraged the adoption of healthier lifestyles. However, the findings of the food intake assessment suggest that, for most of the nutrients analyzed, patients with MS are exposed to the dietary risks due to the many inadequacies in terms of consumption of macro- and micronutrients.

The mean energy intake of the patients in the present cohort was lower than that found by another study of adult Brazilians [17], and the percentage macronutrient intake was similar to those of healthy individuals from Northeast Brazil [11, 36]. The mean percentage protein intake exceeded the recommended level in both men and women. A high protein intake may affect kidney function, changing the glomerular filtration rate; moreover, since MS per se affects kidney function, we detected inadequate micronutrient intake that should be addressed in the clinical follow-up of these patients [37, 38].

Among dietary factors, dietary fiber intake could play an interesting role in the management of MS. In Brazil, fiber intake was found to be insufficient according to the 2008–2009 Household Budget Survey carried out to estimate the household availability of fiber [39]. Despite methodological differences, mean intake of dietary fiber in the current study of 14.3 g/day was similar to the 14.7 g/day reported in a survey conducted in northeast Brazil [39]. This suggests that current fiber intake in Brazil is inadequate: this is a cause for concern, since this nutrient plays a preventive role against several chronic diseases.

Inadequate fiber intake may hinder regulation of body weight, worsen dyslipidemia, and increase blood pressure and insulin resistance, consequently aggravating MS components [40]. The intestinal microbiota is an important factor in the development of obesity and metabolic disorders because of its interactions with the environment (e.g. diet) and/or genetic factors. Hence, dietary strategies to manipulate the intestinal microbiota, such as the use of probiotics and prebiotics, have been proposed as a treatment for obesity and a control for MS [41].

The prevalence of inadequate vitamin D, calcium, and magnesium intakes found by the present study is corroborated by other robust Brazilian studies [17, 36]. The results are worrisome, since patients with MS exhibit inadequate intake of nutrients involved in regulatory and antioxidant mechanisms of action, in conjunction with metabolic risks such as high blood pressure [42] and changes in fasting and postprandial blood glucose levels [43]. In adults, deficiency of 25(OH)D has been negatively linked to traditional MS components, such as waist circumference and triglyceride levels, and non-traditional components, such as fasting insulin levels [14]. Two of these components were among the most frequent in our study population, and, given that vitamin D is a modifiable factor in the nutritional treatment of MS, once vitamin D dietary deficiency is detected, it becomes an important variable of nutritional status.

In addition to the role played in bone metabolism, dietary calcium has been linked to a lower risk of developing MS, as it takes part in the regulatory mechanisms of obesity, hypertension, dyslipidemia, and insulin resistance. The adequate intake of calcium, prioritizing the consumption of low-fat dairy products (except cheese), has been positively associated with a decreased incidence of MS, glucose intolerance, and diabetes mellitus type2 [44].

A low dietary magnesium intake has also been associated with risk factors for MS, such as high fasting blood glucose levels, waist circumference, and triglyceride levels [45]. Sang-Yhun et al. [15], based on a meta-regression model of observational studies, showed a statistically significant inverse association between dietary magnesium intake and risk of MS, with an overall estimate of a 12 % reduction in the risk of MS per each 150 mg/day incremental increase in magnesium intake. This fact may be related to magnesium’s role in glucose and lipid metabolism and blood pressure [46], justifying the importance of adequate magnesium intake.

Some pathophysiological mechanisms of MS arise from the imbalance between the formation and the inactivation of reactive oxygen species [47]. Therefore, the adequate dietary ingestion of antioxidant micronutrients such as vitamins A, C and E, selenium and zinc minerals, may be a protective factor to prevent tissue damage arising from oxidative stress [12, 13]. Puchau et al. showed that the dietary total antioxidant capacity may provide an estimation of the risk of development of MS components [48]. However, findings regarding the association between antioxidant intake and the development of MS remain inconsistent. Wei et al. [13] and Motamed et al. [49] did not observe a significant association between the intake of vitamins A and E and the development of MS.

Inadequate intake of less than 50 % was also observed for B-complex vitamins—thiamine, pyridoxine and niacin—in both men and women, with the exception of riboflavin, which showed an inadequate intake of 53 % in men. A study on dietary pattern conducted in Chinese adults showed that the vitamin B group has a potentially beneficial effect in preventing MS [7]. These results prompt new discussions on the function of water-soluble vitamins in the pathophysiological mechanisms of chronic diseases.

Both men and women had inadequate zinc and selenium intakes. Therefore, zinc deficiency may induce abnormal insulin metabolism and oxidative stress. Besides that, zinc deficiency increases peripheral insulin resistance, central obesity, blood pressure, and triglyceride levels, and decreases HDL-c levels, all components that characterize MS [50]. As an antioxidant, selenium protects against the damage caused by oxidative stress because of the functions of selenoproteins. In an adult population, the dietary intake of selenium showed moderate association with MS in the second quartile of distribution [13]. Plasma selenium levels were correlated positively with all the components of MS in Lebanese adults [51].

Our study showed that the UL of sodium intake was exceeded in both men and women. This is worrisome, and, despite the inability to make inferences regarding exposure and outcomes in our study, we observed that 78 % of patients had high blood pressure, one of the most frequent components of MS. The Korea National Health and Nutrition Examination Survey (KNHANES) demonstrated that sodium excretion is positively associated with MS components such as blood pressure, waist circumference, and triglyceride and fasting glucose levels were negatively associated with HDL-c levels [52]. On the other hand, the potassium intake was below the AI. A high sodium intake combined with a low potassium intake increases peripheral vascular resistance, consequently increasing blood pressure [53].

One of the main limitations of the present study is the inherent complexity of food intake assessment. The evaluation of dietary intake is susceptible to random and systematic errors, although in the present study, the HRs were administered by trained nutritionists who took care to minimize errors while collecting and analyzing data. Moreover, the data obtained were subsequently adjusted based on energy and intra-individual variation. Another limitation is the choice of the IPAQ-SF to measure physical activity. The IPAQ-SF has become the most widely used physical activity questionnaire; however, it tends to overestimate the amount of physical activity [54]. In fact, there are currently no perfect gold standard criteria for measuring physical activity. In our study, this variable was important only for the characterization of the participants. Besides that, we used the IPAQ-SF version validated for the Brazilian population.

## Conclusions

The patients with MS had high protein intake, low fiber intake, and high prevalence of inadequate vitamin D, magnesium, and calcium intakes, regardless of the sex. The markedly inadequate intake of various micronutrients may exacerbate complications of MS. These findings indicate the importance of nutritional guidance in the healthcare of individuals exposed to the risks of MS and diabetes mellitus.

## Abbreviations

MS: metabolic syndrome
HDL-c: high-density lipoprotein cholesterol
24-HR: 24-hour recall
BMI: body mass index
EAR: estimated average requirement
UL: tolerable upper intake level
AI: adequate intake
IPAQ-SF: international physical activity questionnaire-short form
